# Direct Enantiomeric Resolution of Betaxolol with Application to Analysis of Pharmaceutical Products

**Published:** 2007-02-06

**Authors:** Mohamed M. Hefnawy, Maha A. Sultan, Mona M. Al-Shehri

**Affiliations:** Department of Pharmaceutical Chemistry, College of Pharmacy, King Saud University, P.O. Box 2457, Riyadh 11451, Saudi Arabia

**Keywords:** Betaxolol, Enantiomeric resolution, Teicoplanin, Pharmaceutical products

## Abstract

A high-performance liquid chromatographic (HPLC) method has been developed for the separation and determination of S- and R-enantiomers of betaxolol in tablets and ophthalmic preparations. Baseline resolution was achieved by using teicoplanin macrocyclic antibiotic chiral stationary phase (CSP) known as Chirobiotic T with fluorescence detection at excitation/emission wavelengths 275/305 nm. The polar ionic mobile phase (PIM) consists of methanol-glacial acetic acid-triethylamine, (100:0.020:0.025, v/v/v) has been used at a flow rate of 1.5 ml/min. All analytes with S-(–)-atenolol as internal standard were conducted at ambient temperature. The method is highly specific where another coformulated compounds did not interfere. The stability of betaxolol enantiomers under different degree of temperature also studied. The results showed that it is stable for at least 7 days at 70°C. The method validated for its linearity, accuracy, precision and robustness. Experimental design was used during validation to evaluate method robustness. Using the chromatographic conditions described, S- and R-betaxolol were well resolved with mean retention times of 11.3 and 12.6 min, respectively. Linear response (r > 0.997) was observed over the range of 10–500 ng/ml of betaxolol enantiomers, with detection limit of 5 ng/ml. The recoveries of S- and R-betaxolol from tablets and ophthalmic preparation ranged from 97.4 to 101.4% and 98.0 to 102.0%, respectively. The mean relative standard deviation (R.S.D.%) for both enantiomers were 1.1–1.4% and 1.3–1.7% in tablets and ophthalmic solution, respectively.

## Introduction

The determination of enantiomeric purity of pharmaceuticals is subject to severe attention from the clinical and toxicological point of view. Prior to the approval of a new racemic drug, the enantiomers must be analytically and unequivocally separated, and the pharmacological effects as well as the metabolic pathways must be studied separately for each enantiomer [1]. This implies an ever increasing demand for pure enantiomeric compounds and for pertinent enantioselective technologies. Enantiomeric resolutions have acquired an important position in all stages of drug development process. Therefore, the development of new methods for efficient chiral separations and quantitation is more than necessary [2].

In last two decades, high performance liquid chromatography (HPLC) has become one of the mostly applied techniques in the chiral separation of different racemates [3,4]. Several chiral stationary phases (CSPs) have been developed and used for the chiral separation of a variety of racemates. Among these CSPs, macrocyclic glycopeptide antibiotic-based CSP are very important as they have achieved a great reputation in the field of chiral separation. The importance of this type of CSP includes its ease of use, reproducible results and a wide range of applications [5–7].

Macrocyclic antibiotics have been introduced by Armstrong et al. as powerful chiral selector [8–10]. The glycopeptides macrocyclic antibiotics such as teicoplanin and vancomycin have been widely used as a CSP and a great variety of racemic compounds have been resolved on them [11,12]. The enantioselectivity of these chiral selectors due to several reasons: (a) they are amphoteric (i.e. contain acidic and basic ionizable groups); (b) they have the necessary geometry and functionalities that accentuate chiral recognition in solution; and (c) they contain both hydrophilic and hydrophobic moieties [13].

The possible bonding between the enantiomers and the macrocyclic glycopeptide antibiotic CSP has been reviewed [14]. The most important bonding involved is π-π complexation, hydrogen bonding, inclusion complexation, dipole interactions, steric interactions and ionic and cationic bindings. These bonding are a result of the complex structures of this CSP, which consists of sugar moieties, phenyl, quinoline and thiazole rings, along with several chiral centers, inclusion baskets, hydrogen donor and acceptor sites. It has been reported that these bonding sites are responsible for the surprising chiral selectivities of these antibiotics [6,8].

Betaxolol hydrochloride, 1-[4-[2-(cyclopropyl methoxy) ethyl]-phenoxy]-3-[ (1 -methylethyl)amino]-2-propanol, is a cardioselective β-adrenergic antagonist. It exhibits high and consistent bioavailability (70–90%) and a long terminal half-life of (13–20 h) [15]. The drug, marketed as a racemic mixture, is highly efficacious for the treatment of hypertension and glaucoma [16].

The resolution of betaxolol enantiomers was first reported using a lengthy derivatization with R(–)-naphthylethylisocyanate by reversed-phase HPLC and fluorimetric detection[17]. A direct method for the enantiomeric separation of betaxolol on Chiralcel OD column has been developed using UV detection [16]. The separation of betaxolol on a cellulose tris(4-methylbenzoate) chiral stationary phase without derivatization via normalphase HPLC was also reported [18].

This method is first direct reversed-phase chiral HPLC assay in pharmaceutical products utilizing teicoplanin CSP and fluorimetric detection. The method is highly selective where other co-formulated substances did not interfere in the determination.

## Experimental

### Chromatographic conditions

The HPLC instrument (Jasco, Japan) equipped with a pump (model PU-980), a fluorescence detector (model FP-920), a 20 μl injector and the instrument is connected to LG computer. The CSP used in this study was the macrolide-type antibiotic teicoplanin, known as Chirobiotic T (150 × 4.6 mm i.d.) purchased from Advanced Separation Technologies (Whippany, NJ, U.S.A.). The mobile phase was methanol-glacial acetic acid-triethylamine (100:0.020:0.025, v/v/v). The mobile phase was filtered through a Millipore membrane filter (0.2 μm) from Nihon, Millipore (Yonezawa, Japan) and degassed before used. The flow rate was 1.5 ml/min and the detection wavelengths (FL) were set at 275 nm for excitation and 305 nm for emission.

### Chemicals

Betaxolol hydrochloride, S-betaxolol and R-betaxolol were obtained from RBI (Natick, MA, U.S.A.). S-(–)-Atenolol was obtained from Sigma Chemical Co. (St Louis, MO, U.S.A.). HPLC-grade methanol, analytical grade triethylamine and glacial acetic acid were purchased from BDH Chemicals (Poole, U.K). Kerlone^®^ 20 mg tablet (containing 20 mg of betaxolol hydrochloride/tablet) was obtained from Laboratoires Synthelabo (Le Plessis-Robinson/France) and Betoptic^®^ 0.5% drop (containing 0.5% betaxolol hydrochloride and 0.01% benzalkonium chloride) was obtained from Laboratoires Alcon (Rueil-Malmaison Cedex, France).

### Preparation of standard stock solutions

Stock solution containing 1 mg/ml of S- and R-betaxolol were prepared in methanol and serial dilutions with the same solvent were made to cover the working range. The internal standard S-(–)-atenolol was prepared in methanol to give a concentration of 1mg/ml. The solutions were kept in a refrigerator.

### Preparation of standard solutions of tablets

Ten tablets were ground and powdered, an accurately weighed portion equivalent to 20 mg betaxolol was transferred to 100 ml volumetric flask diluted to the mark with methanol. The solution was sonicated for 15 min, centrifuged at 3000 rpm for 10 min. Accurately measured aliquots of the supernatant were transferred to 5 ml volumetric flasks containing 70 μl of the internal standard and diluted to 5 ml with methanol to give final concentration of 50, 250 and 400 ng/ml of betaxolol.

### Preparation of standard solution of ophthalmic drops

The content of ten ophthalmic solutions (0.5%) were mixed well and 1 ml was transferred into 100 ml volumetric flask and diluted to the mark with methanol. Appropriate dilution with the same solvent was made to provide a solution containing 500 ng/ml of betaxolol. Accurately measured aliquots of this solution were transferred to 5 ml volumetric flask containing 70 μl of the internal standard and diluted to 5 ml with methanol to give final concentration of 50, 250 and 400 ng/ml of betaxolol.

### Linearity

Aliquot volumes of the final solution of S(–)- and R(+)-betaxolol were transferred to a series of 5 ml volumetric flasks to produce solutions covering the concentration range from 10–500 ng/ml. A volume equivalent to 70 μl of S-(–)-atenolol was added to each flask and the solution was diluted to 5 ml with methanol. A 20 μl of each standard solution was injected into the chromatographic system and the chromatograms were recorded. Calibration standards of each concentration were analyzed in triplicate. Calibration curves of betaxolol enantiomers were constructed using normalized drug/internal standard peak area ratio versus nominal concentrations of the analyte. Least squares linear regression analysis of the data gave slope, intercept, and correlation coefficient data. The corresponding regression equations were derived for the analyte.

### Quantitation

A 20 μl of the selected assay solutions of tablets and ophthalmic drops were injected into the chromatographic system and the chromatograms were recorded. The nominal contents of the drug in each formulation were calculated from the linear regression equations. The recovery and the RSD were calculated.

### Specificity

The specificity of the method was investigated by observing any interference encountered from coformulated benzalkonium chloride in ophthalmic solution and other excipients present in the tablets.

### Stability of ophthalmic solutions

The stability of ophthalmic solutions was tested by the developed HPLC method over a period of 7 days. The freshly prepared and the 7 day-stored samples at room temperature, 30, 50 and 70°C were analyzed by the proposed HPLC method. The concentrations of the stored samples were calculated and compared to that of the freshly prepared samples.

### Validation

The limit of detection (LOD) and the limit of quantification (LOQ) were determined as 3 and 10 times the baseline noise, respectively [19]. The results of the statistical analysis of the experimental data, such as the slopes, the intercepts, the correlation coefficients obtained by the linear squares treatment of the results along with standard deviation of the slope (S_b_) and intercept (S_a_) on the ordinate and the standard deviation of the residuals (S_y/x_) was shown. The good linearity of the calibration graphs and the negligible scatter of experimental points are clearly evident by the values of the correlation coefficient and standard deviation. The robustness of the method is demonstrated by the versatility of the experimental factors that affect the peak area.

## Results and discussion

**Fig. 1.**
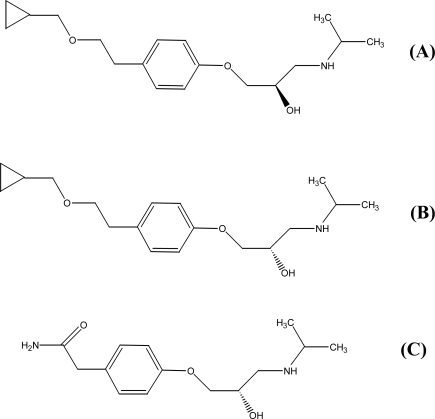
The chemical structure of (**A**) S-betaxolol, (**B**) R-betaxolol and (**C**) S-(–)-atenolol (IS).

The HPLC method carried out in this study, aimed at developing a chromatographic system, capable of eluting and resolving betaxolol enantiomers from pharmaceutical preparations (Fig. 1). The use of methanol-based mobile phases containing different triethylamine and acetic acid ratios was investigated for the separation of enantiomers of betaxolol on teicoplanin column. The best results in terms of resolution, analysis time and separation factor were obtained with mobile phase consisted of methanol-glacial acetic acid-triethylamine (100:0.020:0.025, v/v/v) (Table1). No enantioseparation were observed in the absence of triethylamine. This could be explained on the basis of strong repulsive effects between the protonated amino groups of the analyte molecules and of the CSP. An increase of the triethylamine concentration in the mobile phase (to about 0.1%) decreased the retention factors of the studied analytes. Increasing the concentration of acetic acid in the mobile phase (to about 0.1%) also decreased the retention factors of the studied analytes. This demonstrates that it is the concentration of acetic acid and triethylamine in mobile phase that has a substantial influence on the retention factors and not the ionic strength of the mobile phase that was constant.

Macrocyclic antibiotic chiral stationary phases have been widely used for enantiomers resolution because they are very effectively for the enantioseparation of anionic compounds. The selectivity towards these compounds is because of the presence of amine groups in the chiral selector [20]. If the compound has more than one functional group capable of interacting with the stationary phase and at least one of those groups is on or near the stereogenic center, then the first mobile phase choice would be the polar ionic mobile phase (PIM). Due to the strong polar groups present in the macrocyclic peptides, it was possible to convert the original mobile phase concept to 100% methanol with an acid/base added to effect selectivity [20]. The key factor in obtaining complete resolution is still the ratio of acid to base [5]. The importance and superiority of macrocyclic antibiotics as chiral selector, in comparison with other chiral selector, is because they can be used in normal and reversed phases with greater stability and capacity [21]. The polar ionic mobile phase (PIM) has been described as a developed method to obtain difficult enantioselective separation with macrocyclic antibiotic-based CSP [22]. This approach uses a non-aqueous polar component (methanol) with both glacial acetic acid and triethylamine, which are necessary to achieve enantioseparation.

The linear regression analysis of betaxolol enantiomers in pure solution was constructed by plotting the peak area ratio of each enantiomer to the internal standard (*y*) versus analyte concentration in ng/ml (*x*). The calibration curves were linear in the range of 10–500 ng/ml, with a correlation coefficient (r) of 0.996 for both enantiomers (Table 2). A typical calibration curve has the regression equation of *y* = 0.005 *x –* 0.002 for (S)-betaxolol and *y* = 0.005 *x –* 0.003 for (R)-betaxolol. The limit of detection (LOD) and the limit of quantitation (LOQ) for each enantiomer were 5 ng/ml and 10 ng/ml, respectively (Table 2). The results of the statistical analysis of the experimental data, such as the slopes, the intercepts and the correlation coefficients obtained by the least squares treatment of the results along with standard deviation of the slopes and intercepts on the ordinate and the standard deviation of the residuals were shown in Table 2. The accuracy of the method was tested by analyzing different concentrations of standard betaxolol enantiomers. The results were expressed as percent recoveries of the particular components in the samples (Table 3). The overall recoveries of betaxolol enantiomers in standard solution by the proposed method were 99.6 and 99.4% for S- and R-betaxolol, respectively, with %RSD of 0.62 for S- and R-betaxolol, respectively, indicating that these values were acceptable.

**Fig. 2.**
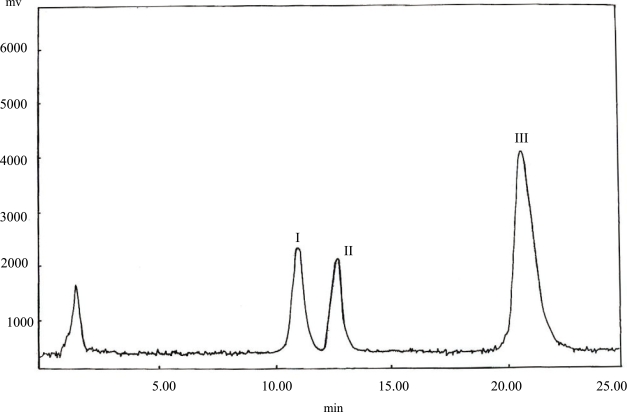
Chromatogram of [I] S-betaxolol,100 ng/ml, [II] R-betaxolol, 100 ng/ml, recovered from Kerlone^®^ 20 mg tablet and/or Betoptic^®^ 0.5% Drop spiked with [III] 10 μg/ml (S)-(–)-atenolol (IS).

The validity of the method developed here was applied to various concentrations taken from the pharmaceutical formulations (Kerlone^®^ 20 mg tablet, and Betoptic^®^ 0.5% drop) for determining their content of betaxolol enantiomers. The values of the overall drug percentage recoveries and the %RSD values of S- and R-betaxolol are presented in Table 4, indicating that these values are acceptable and the method is accurate and precise. Benzalkonium chloride, a preservative which is coformulated with betaxolol in ophthalmic solution, did not interfere with the determination of betaxolol enantiomers indicating the high specificity of the proposed method. Excipients commonly coformulated with the studied drug in tablets such as magnesium stearate, starch, talk powder and binder did not interfere with the determination of betaxolol, indicating the high selectivity of the method (Fig. 2).

The optimum HPLC conditions set for this method have been slightly modified for samples of betaxolol as a mean to evaluate the method robustness. The small changes made include the flow rate, the detection wavelength, temperature and day. Table 5 shows that the percent recoveries of betaxolol enantiomers were good under most conditions and did not show a significant change when the critical parameters were modified. Considering the modification in the system suitability parameters and the specificity of the method, as well as carrying the experiment at room temperature, would conclude that the method conditions are robust.

The stability of ophthalmic solutions versus temperature was tested by the developed HPLC over a period of 7 days. The freshly prepared and the 7 day-stored samples at room temperature, 30, 50 and 70°C were analyzed by the proposed HPLC method. The concentrations of betaxolol enantiomers in the stored samples were calculated and compared to that in freshly prepared samples. There is a non significant difference between the stored and freshly prepared samples, indicating the possibility of using betaxolol solutions over a period of 7 days without degradation.

## Conclusion

A highly specific high-performance liquid chromatographic method has been developed and validated for the separation and quantification of betaxolol enantiomers in tablets and ophthalmic solution. The method utilizes a teicoplanin chiral stationary phase and fluorescence detection. The method is selective for the determination of betaxolol enantiomers in the presence of coformulated benzolkonium chloride without interferences. The total run time for this method is 25 min, which allows processing of over 55 samples per day. This method has provided good sensitivity and excellent precision and reproducibility.

## Figures and Tables

**Table 1.** Chromatographic parameters for betaxolol enantiomers and the internal standard S-(–)-atenolol.

**Analyte**	**R_s_^a^**	**α^b^**	***K*^c,d^**	**T_R_ (min)^d^**
S-betaxolol	1.83	1.12	6.62 ± 0.02	11.36 ± 0.03
R-betaxolol	6.98	1.69	7.46 ± 0.04	12.62 ± 0.05
S-(–)-atenolol	^e^	^e^	12.61 ± 0.01	20.29 ± 0.02

^a^ *R*_s_ = (*t*_2_ – *t*_1_)/0.5(*w*_1_+*w*_2_)

^b^ Separation factor, calculated as *k*_2_/*k*_1_

^c^ Capacity factor, calculated as *T_R_* – *T*_0_/*T*_0_

^d^ Mean ± SD, n = 3

^e^ Not calculated

**Table 2.** Validation parameters for the determination of betaxolol enantiomers using the proposed method.

**Parameter**	**S-betaxolol**	**R-betaxolol**
Concentration range (ng/ml)	10–500	10–500
Intercept (a)	−0.002	−0.003
Slope (b)	0.005	0.005
Correlation coefficient (r)	0.996	0.996
S_y/x_	0.034	0.036
S_a_	0.030	0.031
S_b_	0.0004	0.0005
LOD (ng/ml)^a^	5	5
LOQ (ng/ml)	10	10

^a^ S/N = 3

**Table 3.** Determination of betaxolol enantiomers in standard solutions by the proposed method.

**Analyte**	**Enantiomer**	**Nominal conc.(ng/ml)**	**Measured conc.(ng/ml)**	**Recovery (%)**
Betaxolol	S	50	49.75	99.50
		250	247.25	98.90
		400	401.60	100.40
Overall recovery RSD (%)				99.60
				0.62
	R	50	49.70	99.40
		250	247.25	98.70
		400	400.80	100.20
Overall recovery RSD (%)				99.43
				0.62

**Table 4.** Determination of betaxolol enantiomers in pharmaceutical preparations by the proposed method.

**Pharmaceutical Preparation**	**Enantiomer**	**Nominal conc.(ng/ml)**	**Measured conc.(ng/ml)**	**Recovery (%)**
Kerlone^®^ 20 mg tablets^a^	S	50	49.50	99.00
		250	251.00	100.40
		400	398.40	99.60
Overall recovery RSD (%)				99.66
				0.58
	R	50	49.40	98.80
		250	250.50	100.20
		400	398.80	99.70
Overall recovery RSD (%)				99.56
				0.58

Betoptic^®^ 0.5% ophthalmic solution^b^	S	50	50.48	100.96
		250	248.88	99.55
		400	400.75	100.15
Overall recovery RSD (%)				100.22
				0.58
	R	50	50.43	100.86
		250	248.63	99.45
		400	401.75	100.35
Overall recovery RSD (%)				100.22
				0.58

^a^ Product of Laboratoires Synthelabo (Le Plessis-Robinson/France).

^b^ Product of Laboratoires Alcon (Rueil-Malmaison Cedex, France).

**Table 5.** Effect of experimental parameters on percent recoveries of betaxolol enantiomers.

**Parameters**	**Modification**	**Recovery (%)**	
		**S**	**R**
Flow rate (ml/min)	1.2	99.8	99.6
	1.5	98.6	98.8
	1.8	98.4	98.5
Wavelength of excitation (nm)	272	100.8	100.6
	275	99.6	99.5
	278	100.6	100.5
Wavelength of emission (nm)	302	99.8	100.0
	305	98.9	98.8
	308	100.4	100.6
Temperature^a^ (°C)	30	100.1	100.3
	50	98.6	98.7
	70	97.4	97.6
Day^b^	1	100.5	102.3
	3	99.4	101.4
	5	100.0	99.7
	7	98.8	98.9

^a^ 7-day stored ophthalmic solutions at 30, 50 and 70°C.

^b^ Ophthalmic solutions were stored at room temperature.
